# Superficial ulnar artery diagnosed by vascular ultrasound: case report

**DOI:** 10.1590/1677-5449.202300852

**Published:** 2024-02-05

**Authors:** Mariana Jordão França, Luciana Akemi Takahashi, Graciliano José França, Luiz Fernando Tosi Ferreira

**Affiliations:** 1 Universidade Positivo - UP, Curitiba, PR, Brasil.; 2 Universidade Federal do Paraná - UFPR, Hospital de Clínicas, Curitiba, PR, Brasil.

**Keywords:** Doppler ultrasound, ulnar artery, anatomical variation, case report

## Abstract

The largest branch of the terminal division of the brachial artery is the ulnar artery, which arises after the cubital fossa. This artery usually has a deep path in the muscles of the anterior forearm and is responsible for vascularization of the superficial and deep musculature on the ulnar side of the forearm and hypothenar area of the hand. We report an anatomical variant diagnosed by Doppler ultrasound in which the ulnar artery had a superficial position in the forearm. Occurrence of a superficial ulnar artery is rare, but it is an important fact for clinicians, surgeons, and nursing professionals.

## INTRODUCTION

The arterial anatomy of the upper extremity begins at the subclavian artery, which is a branch of the brachiocephalic trunk on the right and a branch of the aortic arch on the left. It continues as the axillary artery, which starts at the external margin of the first rib and extends to the inferior margin of the teres major muscle. After passing the border of the teres major muscle, it becomes known as the brachial artery. The course of the brachial artery begins medial to the humerus before passing anterior, extending to the end of the elbow joint, and branching into the ulnar and radial arteries at the cubital fossa.^1^

The radial artery follows a lateral and superficial path and can be palpated medial of the styloid process of the radius. After the first dorsal interosseous muscle, the artery crosses the metacarpal bones and forms the principal part of the deep palmar arch and also contributes to formation of the superficial palmar arch.^1^

The ulnar artery follows a medial path in the forearm and is the larger of the terminal branches arising from division of the brachial artery. Usually, the ulnar artery passes deep of the flexor digitorum profundus and gives origin to the common interosseous artery, which branches into the anterior and posterior interosseous arteries. After the flexor retinaculum, the artery forms the principal part of the superficial palmar arch and contributes to formation of the deep palmar arch.^1^

Anatomic variations are common in the upper extremity and can be caused by problems related to vasculogenesis or angiogenesis.^2^ Vascular ultrasound is a diagnostic method that is rapidly available, easily accessed, and of low cost and has considerable capacity for identifying such arterial variations. Diagnosing these variations is very important in many areas of medicine and nursing.

**Figure 1:**
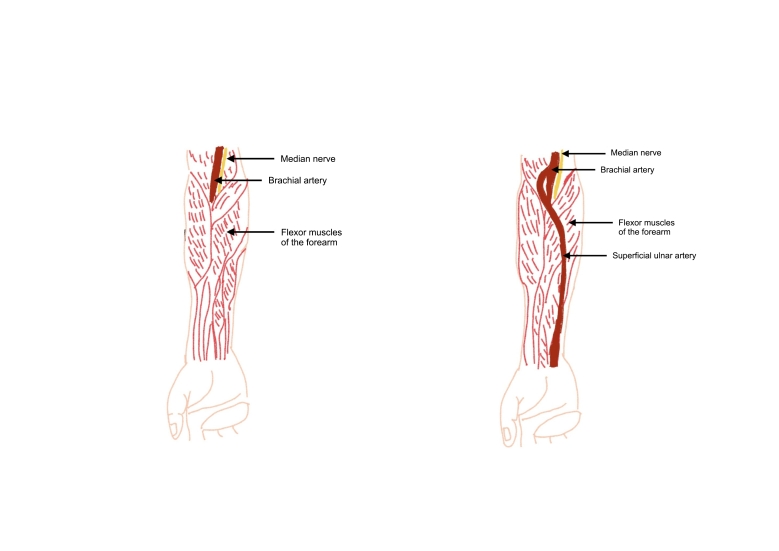
Schematic drawing of the anatomy of the upper limb. The left side of the figure illustrates an ulnar artery with the usual path, below the flexor muscles of the forearm. The right side illustrates an ulnar artery with an unusual path, above the flexor muscles do forearm.

One of the possible anatomic variations of the arteries of the upper extremity is a superficial ulnar artery. In this case, the ulnar artery follows an unusual path, coursing superficial to the flexor muscles of the forearm (Figure 1), and may take origin from the brachial artery, as usual, or even from the axillary artery. The rate of occurrence of cases of superficial ulnar artery varies from 0.7-9.4%^3^ and they are more common in the right upper limb.^4^

This study was approved by the Ethics Committee at our institution (decision number 5.702.204). A free and informed consent form for studies involving human beings was signed.

## CASE DESCRIPTION

**Figure 2:**
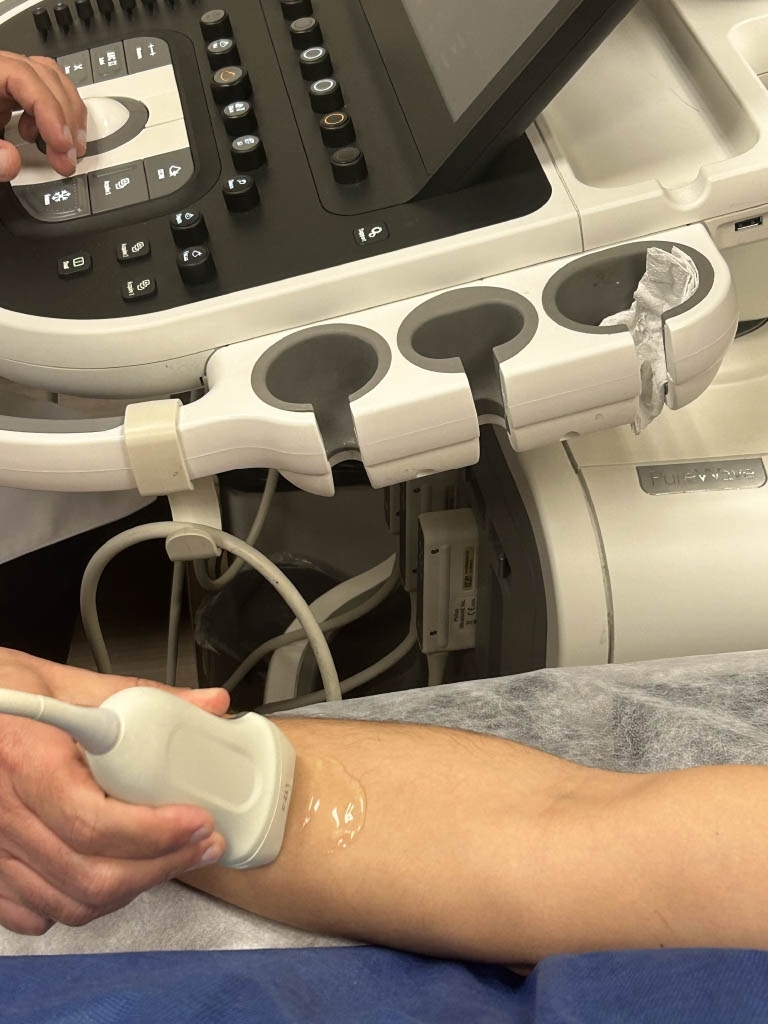
Image illustrating the position of the transducer at the medial forearm of the patient for assessment of the ulnar artery.

**Figure 3:**
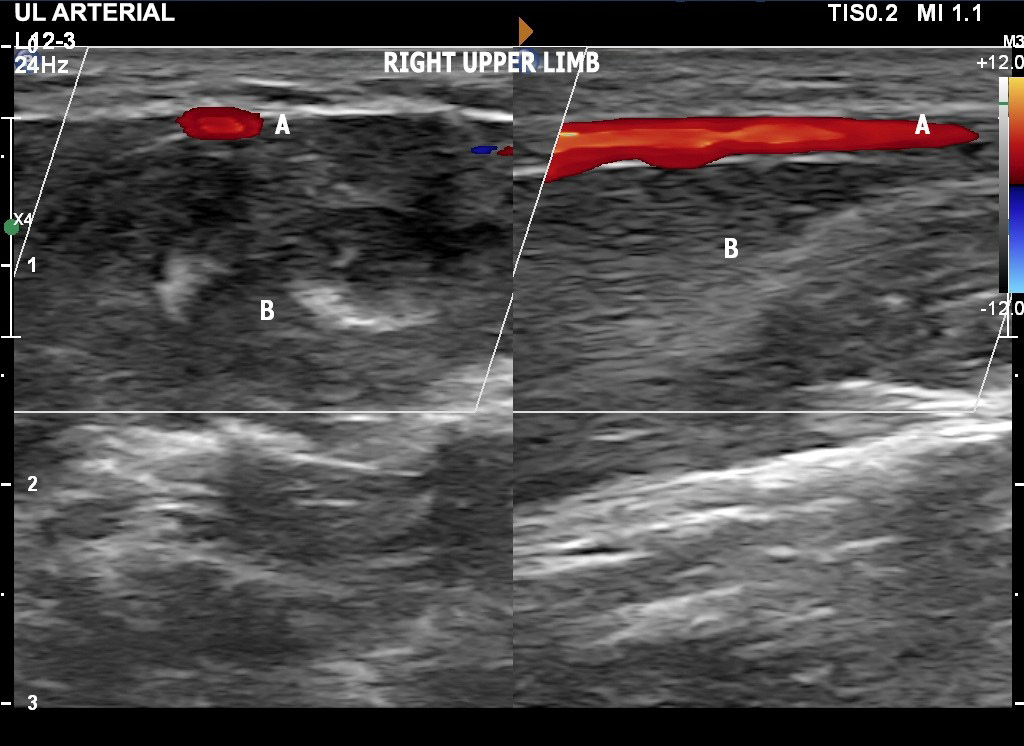
Doppler ultrasonography of the right upper limb showing presence of the superficial ulnar artery anatomic variant, showing the transverse image on the left and the longitudinal image on the right. Labels indicate **(A)** the ulnar artery, in a superficial position, and **(B)** the flexor muscles of the forearm.

**Figure 4:**
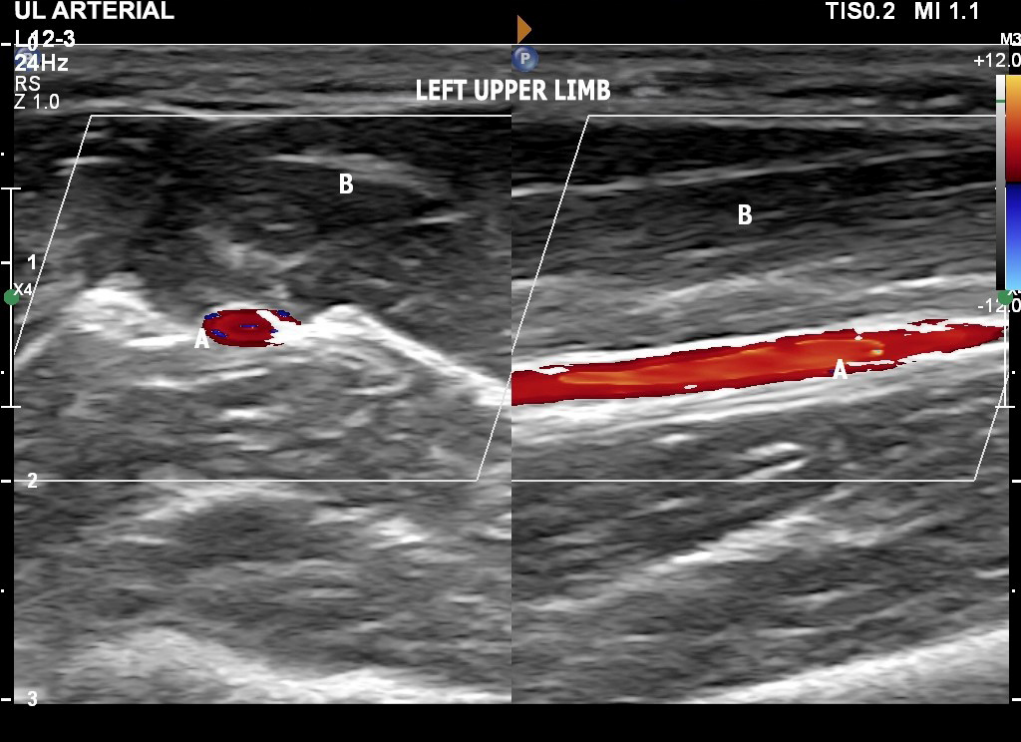
Doppler ultrasound of the left upper limb with the ulnar artery in its normal position, showing the transverse image on the left and the longitudinal image on the right. Labels indicate **(A)** the ulnar artery, in a superficial position, and **(B)** the flexor muscles of the forearm.

The patient was a 30-year-old male physician with no comorbidities, no regular medications, no prior admissions, and no history or traumatism to the right upper extremity. He sought a medical consultation because of a pulse with perceptible amplitude palpable at the medial aspect of the right forearm. Physical examination with palpation of the cubital fossa and the forearm confirmed the presence of a palpable pulse at the medial aspect of the forearm. Arterial vascular ultrasound of the upper limbs was ordered for diagnosis, with the objectives of defining the vascular anatomy, identifying possible anatomic variants, and excluding aneurysmal disease. The examination (Figure 2) was performed using a high frequency linear transducer, with the patient supine, assessing the medial aspect of the forearm and the cubital fossa. Images were acquired in the transverse and longitudinal planes and spectral analysis was performed. Vascular ultrasound ruled out aneurysms and pseudoaneurysms, showing a superficial ulnar artery originating at the brachial artery and with normal dimensions and arterial flow waves according to spectral Doppler (Figure 3). There was no anatomic evidence of a similar variant in the left upper limb (Figure 4). The patient was discharged after the examination by the vascular surgeon and instructed to mention this anatomic variant before any type of medical or nursing procedure involving the right upper limb.

## DISCUSSION

Cases in which the ulnar artery follows a superficial path in the forearm are well-described in the scientific literature. Studies with cadaveric autopsies found prevalence of this anatomic variant in the range of 0.7-9.4%.^3^ A cadaveric study published in 1995 with the objective of reporting anatomic anomalies in the forearm found a superficial ulnar artery in 2.5% of 408 upper limbs studied (204 cadavers).^5^ Other authors have determined the incidence of this rare variant as 0.7-7%.^6-9^ Even though there are few cases, its clinical importance should not be underestimated.

Presence of a superficial ulnar artery does not always impose any disadvantage or risk. Its existence can enable use of flaps with vascularization from the ulnar artery rather than the radial artery, which are normally used when the ulnar artery has its normal deep position. Since the superficial ulnar artery runs above the flexor muscles, flaps for head and neck plastic surgery (Chinese flap) will cause less tendon exposure than the radial flap, resulting in lower rates of complications for the donor. There are also esthetic advantages. First, the skin of the medial aspect of the forearm has less hair, which is an important factor with regard to flaps for the head and neck. Additionally, the ulnar side of the forearm is less visible, which also yields an esthetic advantage, since it means the postoperative scar is less visible.^6^ This is compounded by the fact that the deep palmar arch, which is predominantly formed by the radial artery, is complete in the majority of patients (97%), whereas the superficial palmar arch, predominantly formed by the ulnar artery, is complete in 79% of patients.^10^ As such, in patients with a superficial ulnar artery, use of an ulnar flap is preferable to using a radial flap, maintaining a complete deep palmar arch and guaranteeing normal arterial supply to the thenar and hypothenar areas of the hand.

Nevertheless, this anatomic variant makes the ulnar artery more susceptible to traumas and resulting hemorrhages, since it is not protected by the flexor muscles of the forearm.^11^ It is important that patients know when they have this anatomic variant, so they can inform health professionals who are treating them about the risk of inadvertent puncture of the superficial ulnar artery when drawing blood or administering peripheral venous infusions, primarily when attempting to puncture the basilic veins in the forearm or the intermediate vein in the elbow.^12,13^

**Figure 5:**
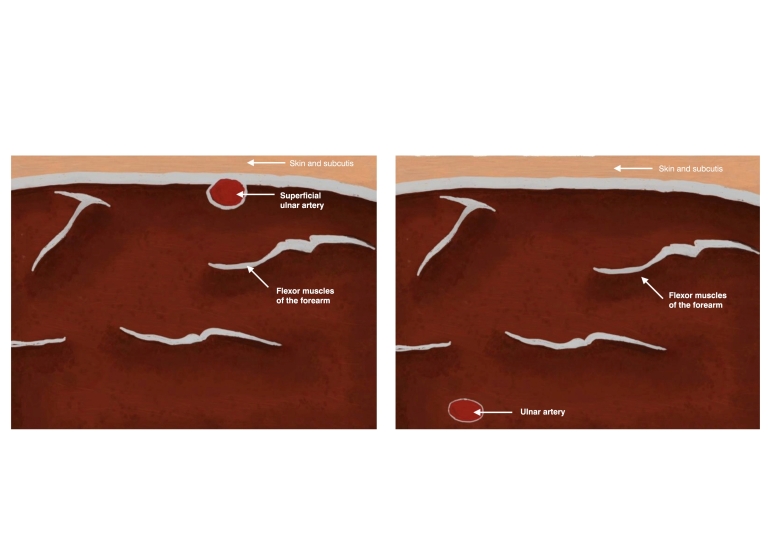
Schematic drawing showing, on the left, the superficial ulnar artery and, on the right, the normal path of the ulnar artery. The skin and subcutis are shown in pale pink and, in cross-section, the superficial ulnar artery coursing above the flexor muscles of the forearm, shown in red.

Finally, this arterial variant also increases the likelihood of an ulnar artery injury during surgery involving the forearm. To avoid such injuries, it is always important to bear in mind the possibility of vascular anatomic anomalies before any procedure. During clinical examination, suspicion of a diagnosis of superficial ulnar artery involves correct palpation of the cubital fossa and forearm.^6^ However, this clinical test cannot definitively confirm a superficial ulnar artery and supplementary diagnostic tests are needed.^3^ In such scenarios, vascular ultrasonography, as a widely available, rapid, and inexpensive noninvasive method is the preoperative examination of choice in patients with a clinical suspicion of superficial ulnar artery (Figure 5).^6,14^

Possible vascular ultrasonography findings include a superficial ulnar artery with a high origin from the brachial artery in the arm^5^ or even with origin from the axillary artery.^15^

In our case, the superficial ulnar artery originated in the cubital fossa in front of the neck of the radius, following a superficial path along the medial aspect of the forearm above the flexor muscles, and with caliber within normal limits compared to the non-superficial contralateral ulnar artery.

## CONSENT FORM

The patient signed a free and informed consent form permitting publication of the ultrasonographic images and case description.
