# Supplementary material for: Superficial ulnar artery diagnosed by vascular ultrasound: case report
Source: J Vasc Bras. 2024 Feb 5;23:e20230085. doi: 10.1590/1677-5449.202300852 (PMC10903516; doi:10.1590/1677-5449.202300852)
Supplement: Translation

# Translation: Artéria ulnar superficial diagnosticada por ultrassom vascular: relato de caso

**DOI:** 10.1590/1677-5449.202300851

**Authors:** Mariana Jordão França, Luciana Akemi Takahashi, Graciliano José França, Luiz Fernando Tosi Ferreira

**Affiliations:** 1 Universidade Positivo - UP, Curitiba, PR, Brasil.; 2 Universidade Federal do Paraná - UFPR, Hospital de Clínicas, Curitiba, PR, Brasil.

**Keywords:** ultrassom Doppler, artéria ulnar, variação anatômica, relato de caso

## Abstract

O maior ramo da divisão terminal da artéria braquial é a artéria ulnar, que se origina após a fossa cubital. Essa artéria usualmente tem trajeto profundo aos músculos do antebraço anterior e é responsável pela vascularização da musculatura superficial e profunda da região ulnar do antebraço e hipotênar da mão, sendo a principal responsável pela formação do arco palmar superficial após o retináculo dos flexores. Reportamos uma variação anatômica após diagnóstico com ultrassom vascular na qual a artéria ulnar situava-se em posição superficial no antebraço. A ocorrência da artéria ulnar superficial é rara, porém de grande importância para clínicos, cirurgiões e profissionais de enfermagem.

## INTRODUÇÃO

A anatomia arterial do membro superior se inicia com a artéria subclávia, a qual é um ramo do tronco braquiocefálico à direita e um ramo do arco aórtico à esquerda. Sua continuação é a artéria axilar, a qual se inicia na borda externa da primeira costela e se estende até a borda inferior do músculo redondo maior. Após passar a margem do músculo redondo maior, passa a ser denominada de artéria braquial. A artéria braquial inicia com trajeto medial ao úmero e torna-se anterior, se estende até o fim da articulação do cotovelo, e na fossa cubital se ramifica em artéria ulnar e radial^1^ .

A artéria radial segue trajeto lateral e superficial, podendo ser palpada medialmente ao processo estiloide do rádio. Após o primeiro músculo dorsal interósseo, a artéria cruza os ossos do metacarpo e forma a parte principal do arco palmar profundo, além de contribuir para formar o arco palmar superficial^1^ .

A artéria ulnar possui trajeto medial no antebraço, sendo o maior ramo terminal da divisão da artéria braquial. Usualmente a artéria ulnar tem trajeto profundo ao músculo flexor profundo dos dedos. Além disso, dá origem à artéria interóssea comum, que se ramifica em interóssea anterior e posterior. Após o retináculo dos flexores, a artéria forma a parte principal do arco palmar superficial e contribui para a formação do arco palmar profundo^1^ .

Variações anatômicas no membro superior são frequentes e podem ocorrer devido a problemas na vasculogenênese ou angiogênese^2^ . Um método diagnóstico disponível rápido, de fácil acesso, baixo custo e grande capacidade de localização dessas variações arteriais é o ultrassom vascular. Diagnosticar essas variações é de grande importância em diversas áreas médicas e de enfermagem.

Uma das variações anatômicas que podem ocorrer nas artérias do membro superior é a artéria ulnar superficial. Nesse caso, a artéria ulnar tem trajeto não usual, sendo superficial aos músculos flexores do antebraço (Figura 1), podendo ter origem usual a partir da artéria braquial, ou até mesmo da artéria axilar. A ocorrência de casos de artéria ulnar superficial varia entre 0,7-9,4%^3^ , sendo mais comum no membro superior direito^4^ .

**Figura 1:** Desenho esquemático da anatomia do membro superior. No lado esquerdo da figura está representada uma artéria ulnar com trajeto usual abaixo dos músculos flexores do antebraço. No lado direto, está representada uma artéria ulnar com trajeto não usual acima dos músculos flexores do antebraço.

O estudo foi aprovado pelo Comitê de Ética de nossa instituição (parecer número 5.702.204). O termo de consentimento livre e esclarecido foi assinado para os estudos que envolvem seres humanos.

## DESCRIÇÃO DO CASO

Homem de 30 anos, médico, sem comorbidades, não fazia uso de medicações regulamente, negou internações prévias e referiu nunca ter sido vítima de traumatismos em membro superior direito. Procurou avaliação médica devido a pulso palpável e com percepção de amplitude na face medial do antebraço direito. No exame físico foi realizada a palpação da fossa cubital e do antebraço, confirmando a presença de um pulso palpável na face medial do antebraço. Para o diagnóstico, foi solicitado ultrassom vascular arterial dos membros superiores, com o objetivo de definir a anatomia vascular, identificar possível variação anatômica e excluir doença aneurismática. O exame (Figura 2) foi realizado com transdutor linear de alta frequência, com o paciente em decúbito dorsal, a fim de avaliar a face medial do antebraço e a fossa cubital. Foram realizados cortes transversais, longitudinais e análise espectral. O ultrassom vascular excluiu a possibilidade de aneurisma ou pseudoaneurisma, revelando artéria ulnar superficial com origem a partir da artéria braquial, apresentando dimensões e onda arterial normais pelo Doppler espectral (Figura 3). Não havia evidência anatômica de variação similar no membro superior esquerdo (Figura 4). O paciente foi liberado após avaliação do cirurgião vascular e orientado a informar essa variação anatômica para qualquer procedimento médico ou de enfermagem (venopunção) no membro superior direito.

**Figura 2:** Imagem demonstrando posição do transdutor na parte medial do antebraço do paciente para avaliar a artéria ulnar.

**Figura 3:** Eco-Doppler do membro superior direito com a presença da variação anatômica da artéria ulnar superficial, demonstrando à esquerda o corte transversal e à direita o corte longitudinal. Está indicado em **A)** artéria ulnar, a qual tem percurso superficial, e **B)** músculos flexores do antebraço.

**Figura 4:** Ecodoppler do membro superior esquerdo com a artéria ulnar em seu trajeto usual, demonstrando à esquerda o corte transversal e à direita o corte longitudinal. Está indicado em **A)** artéria ulnar, e **B)** músculos flexores do antebraço.

## DISCUSSÃO

Casos em que a artéria ulnar tem trajeto superficial no antebraço estão bem reportados cientificamente. Estudos prévios realizados em cadáveres submetidos a autópsia encontraram uma prevalência dessa variação anatômica entre 0,7-9,4%^3^ . Um estudo cadavérico realizado em 1995 com o objetivo de encontrar alterações anatômicas no antebraço reportou que, dos 408 membros superiores estudados (204 cadáveres), a artéria ulnar superficial foi encontrada em 2,5% dos casos^5^ . Já outros autores identificaram que essa rara variação tem incidência entre 0,7-7%^6-9^ . Mesmo com poucos casos, sua importância clínica não deve ser subestimada.

A presença da artéria ulnar superficial nem sempre pode significar uma desvantagem ou risco. Sua presença pode permitir a utilização de retalhos com vascularização da artéria ulnar em vez da artéria radial, usada mais comumente em casos de artéria ulnar com trajeto habitual profundo. Pelo fato de a artéria ulnar superficial ter seu trajeto acima dos músculos flexores, em retalhos de cirurgia plástica para face e pescoço (retalho chinês) haverá menor exposição de tendões comparado ao retalho radial, o que leva a menor taxa de complicação para o doador. Outrossim, há vantagens estéticas. Primeiramente, a pele do lado medial do antebraço apresenta menor pilificação, o que é um fator importante em se tratando de retalhos para cabeça e pescoço. Além disso, o lado ulnar do antebraço é menos visível, o que representa uma vantagem estética, por deixar a cicatriz pós-operatória menos visível^6^ . Soma-se a isso o fato de o arco palmar profundo, predominantemente formado pela artéria radial, ser completo na maior parte dos casos (97% dos pacientes) comparativamente ao arco palmar superficial, majoritariamente formado pela artéria ulnar (completo em 79% dos pacientes)^10^ . Dessa maneira, em pacientes com artéria ulnar superficial é preferível usar retalho ulnar a retalho radial, deixando assim o arco palmar profundo completo e garantindo o suprimento arterial normal da mão para a região tenar e hipotenar.

Ainda, essa variação anatômica torna a artéria ulnar mais suscetível a traumas e consequentes hemorragias, já que não existe a proteção dos músculos flexores do antebraço^11^ . É importante que os pacientes tenham conhecimento da presença dessa variação anatômica, para que possam alertar os profissionais de saúde que estejam lhes atendendo quanto ao risco de punção inadvertida da artéria ulnar superficial em eventuais coletas de sangue ou nas infusões venosas periféricas, principalmente ao se tentar puncionar as veias basílica no antebraço ou intermédia do cotovelo^12,13^ .

Finalmente, essa variação arterial também aumenta a possibilidade de lesão da artéria ulnar durante cirurgia do antebraço. Para evitar essas lesões, é sempre importante ter em mente a possibilidade de alterações anatômicas vasculares antes de qualquer procedimento. Durante o exame clínico, a suspeita do diagnóstico de artéria ulnar superficial envolve palpação correta da fossa cubital e do antebraço^6^ . Todavia, o teste clínico não pode afirmar com certeza a ocorrência da artéria ulnar superficial, sendo necessária complementação diagnóstica^3^ . Nesse cenário, a ultrassonografia vascular, por ser um método não invasivo, amplamente disponível, rápido e de baixo custo, é o exame de escolha pré-operatório nos pacientes com suspeita clínica de artéria ulnar superficial (Figura 5)^6,14^ .

**Figura 5:** Desenho esquemático demonstrando à esquerda o trajeto da artéria ulnar superficial e à direita o trajeto da artéria ulnar usual. Em rosa claro está sinalizado a pele e o tecido subcutâneo, em corte transversal a artéria ulnar superficial com trajeto acima dos músculos flexores do antebraço, demonstrados no desenho em vermelho.

Como achados na ultrassonografia vascular podemos ter uma artéria ulnar superficial com origem mais alta na artéria braquial no braço^5^ , ou até mesmo origem pela artéria axilar^15^ .

Em nosso caso, a artéria ulnar superficial apresentava origem na fossa cubital defronte ao colo do rádio, seguindo percurso superficial na face medial do antebraço acima dos músculos flexores, e com calibre dentro da normalidade comparada com a artéria ulnar contralateral não superficial.

## TERMO DE CONSENTIMENTO

O paciente assinou o termo de consentimento livre e esclarecido para a divulgação de imagens ultrassonográficas e publicação do caso.
